# Full-Length 16S rRNA Gene Sequences from Raw Sewage Samples Spanning Geographic and Seasonal Gradients in Conveyance Systems across the United States

**DOI:** 10.1128/mra.00319-22

**Published:** 2022-06-21

**Authors:** Emily Lou LaMartina, Angela L. Schmoldt, Ryan J. Newton

**Affiliations:** a School of Freshwater Sciences, University of Wisconsin-Milwaukee, Milwaukee, Wisconsin, USA; b Great Lakes Genomics Center, Milwaukee, Wisconsin, USA; University of Southern California

## Abstract

Wastewater microbiome research often relies on sequencing of hypervariable regions of 16S rRNA genes, which are difficult to classify at refined taxonomic levels. Here, we introduce a data set of near-full-length 16S rRNA genes from samples designed to capture known geographic and seasonal variations in municipal wastewater microbial communities.

## ANNOUNCEMENT

Wastewater-based monitoring for disease-causing entities is growing as a public health tool (1, 2). However, there remain significant gaps in understanding the inherent biology of sewage conveyance and its potential influence on monitoring efforts. To aid the characterization of wastewater microorganisms, 46 raw wastewater treatment plant (WWTP) influent samples underwent near-full-length 16S rRNA gene sequencing. We selected samples that, according to previous work, encompass microbial community variability across geographic and seasonal gradients (3, 4). Raw influent (25-mL) samples were filtered onto 0.2-μm mixed cellulose ester filters (product number WHA10401770; MilliporeSigma), from which DNA was extracted with the FastDNA Spin kit for soil (product number 116560200-CF; MP Biomedicals) as described previously (3, 4). Genes were amplified using KAPA HiFi HotStart ReadyMix (product number KK2602; Roche) with the primers 27F (5′-AGRGTTYGATYMTGGCTCAG-3′) and 1492R (5′-RGYTACCTTGTTACGACTT) under the following thermocycler conditions: 95°C for 5 min; 20 cycles of 98°C for 20 s, 55°C for 45 s, and 72°C for 3 min; and 72°C for 5 min. Each primer contained a pad sequence (GGTAG) followed by a unique 16-bp barcode appended to the 5′ end. Prior to PCR, the barcoded primers were phosphorylated with a T4 polynucleotide kinase (product number M0201S; New England Biolabs) and ATP (product number P0756S; New England Biolabs).

Following PCR, amplicons were equimolarly pooled and purified with AMPure PB beads (product number 100-265-900; Pacific Biosciences [PacBio]). Libraries were created using the SMRTbell Express Template 2.0 (product number 101-685-400; PacBio) following the manufacturer’s protocol. Amplicons were enzymatically repaired and ligated to a PacBio adapter to form the SMRTbell template. Templates were sequenced on a Sequel II system using sequencing primer v.4 (product number 101-359-000; PacBio) and the Sequel II 2.1 binding kit (product number 101-820-500; PacBio). The University of Wisconsin-Milwaukee Great Lakes Genomics Center (Research Resource Identifier [RRID] SCR_017838) provided PacBio sequencing services.

Default parameters were used for all software unless otherwise specified. BAM files from the PacBio Sequel II system were converted to FASTQ files with BEDtools v.2.30.0 (5). SeqKit v.2.2.0 (6) was used to demultiplex FASTQ files into individual files according to their unique barcodes. Primers were removed from demultiplexed files with Cutadapt (7). Following a PacBio-specific protocol, DADA2 v.1.16 (8) on Galaxy v.22.01 (9) was used to quality filter (maximum *N* = 0, maximum EE = 2), correct errors, and assign taxonomy with SILVA v.138 (10) as a reference database. For most reads, the first 10 primer bases on the 3′ end of the read were not trimmed by Cutadapt. These bases were removed with an exact-match approach (grep/cut). The resulting amplicon sequence variants (ASVs) were clustered to operational taxonomic units (OTUs) at 99.5% similarity with mothur v.1.43.0 (11) and its protocol (https://mothur.org/wiki/cluster).

**FIG 1:**
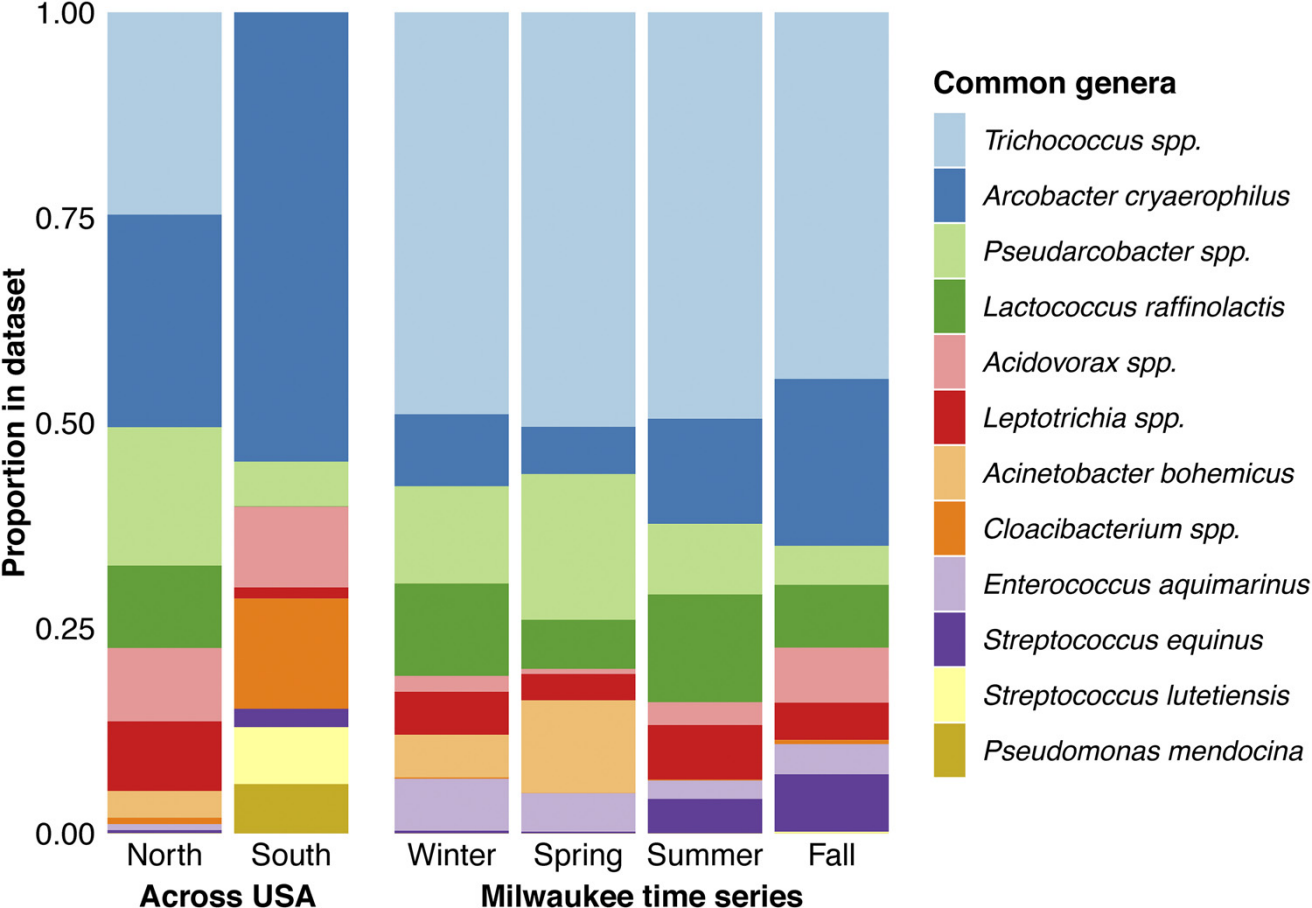
Genus and species assignments of abundant OTUs. The top 5 most abundant OTUs in each wastewater sample set (north and south United States and winter, spring, summer, and fall in Milwaukee, Wisconsin) were identified, comprising 64.0% of all sequences. Bar height indicates the proportion of that OTU among the common OTUs analyzed. Bar colors denote genus and species assignments of OTUs.

Before demultiplexing, the raw FASTQ file had 7,750,870 reads, which were condensed to 1,041 ASVs and 698 OTUs. A summary of raw, ASV, and OTU data is presented in Table 1. ASVs ranged from 1,383 to 1,553 bp, with a mean length of 1,455 bp. All ASVs were classified as bacteria and included 22 phyla, 35 classes, 71 orders, 116 families, 190 genera, and 158 species. See Fig. 1 for the most abundant OTUs. The improved taxonomic resolution from full-length gene sequences resulted in 643 ASVs (61.8%) classified to the species level, compared to 3.48% in a V4-V5 hypervariable region study of a similar sample set (4).

**TABLE 1:** Summary of demultiplexed sequencing data (BioProject accession number PRJNA809416)

BioSample accession no.	SRA accession no.	State of sample collection	Date (yr-mo-day)	No. of raw reads	No. of ASVs	No. of OTUs
SAMN26027580	SRR18111974	Montana	2013-1-16	8,124	29	25
SAMN26027581	SRR18111973	Oregon	2013-1-16	50,475	66	38
SAMN26027582	SRR18111962	Washington	2013-1-15	39,857	86	63
SAMN26027583	SRR18111951	Iowa	2013-1-15	48,102	73	57
SAMN26027584	SRR18111940	Nebraska	2013-1-23	49,510	63	39
SAMN26027585	SRR18111933	Wisconsin	2013-1-27	61,322	90	58
SAMN26027586	SRR18111932	Alaska	2013-1-23	44,505	89	65
SAMN26027587	SRR18111931	Wyoming	2013-1-24	40,151	37	21
SAMN26027588	SRR18111930	Colorado	2013-1-23	63,240	74	44
SAMN26027589	SRR18111929	Texas	2012-8-30	50,787	116	98
SAMN26027590	SRR18111972	Alabama	2012-8-14	38,960	70	55
SAMN26027591	SRR18111971	Georgia	2012-8-16	48,628	78	60
SAMN26027592	SRR18111970	California	2012-8-14	43,504	78	59
SAMN26027593	SRR18111969	Florida	2012-8-8	42,993	87	78
SAMN26027594	SRR18111968	Tennessee	2012-8-15	40,164	66	50
SAMN26027595	SRR18111967	Texas	2012-8-15	40,220	62	49
SAMN26027596	SRR18111966	Arizona	2012-8-15	39,140	62	51
SAMN26027597	SRR18111965	California	2012-8-21	48,423	59	36
SAMN26027598	SRR18111964	Florida	2012-8-21	43,786	151	109
SAMN26027599	SRR18111963	Hawaii	2012-9-7	43,509	57	42
SAMN26027600	SRR18111961	Minnesota	2013-1-16	52,709	58	34
SAMN26027601	SRR18111960	Ohio	2013-1-17	36,980	55	40
SAMN26027602	SRR18111959	Wisconsin	2016-4-7	37,955	83	61
SAMN26027603	SRR18111958	Wisconsin	2017-4-3	44,272	116	84
SAMN26027604	SRR18111957	Wisconsin	2016-8-3	37,141	52	41
SAMN26027605	SRR18111956	Wisconsin	2017-8-22	66,278	84	52
SAMN26027606	SRR18111955	Wisconsin	2016-12-7	53,471	66	43
SAMN26027607	SRR18111954	Wisconsin	2017-12-1	40,778	71	49
SAMN26027608	SRR18111953	Wisconsin	2016-2-8	99,767	95	61
SAMN26027609	SRR18111952	Wisconsin	2017-2-6	42,116	65	45
SAMN26027610	SRR18111950	Wisconsin	2016-1-6	72,965	110	74
SAMN26027611	SRR18111949	Wisconsin	2017-1-5	32,035	71	53
SAMN26027612	SRR18111948	Wisconsin	2016-7-18	46,548	55	40
SAMN26027613	SRR18111947	Wisconsin	2017-7-12	52,143	81	56
SAMN26027614	SRR18111946	Wisconsin	2016-6-8	48,861	55	41
SAMN26027615	SRR18111945	Wisconsin	2017-6-7	45,176	72	46
SAMN26027616	SRR18111944	Wisconsin	2016-3-2	29,799	47	28
SAMN26027617	SRR18111943	Wisconsin	2017-3-1	60,041	69	40
SAMN26027618	SRR18111942	Wisconsin	2016-5-2	40,253	83	64
SAMN26027619	SRR18111941	Wisconsin	2017-5-1	9,838	46	38
SAMN26027620	SRR18111939	Wisconsin	2016-11-3	38,702	58	39
SAMN26027621	SRR18111938	Wisconsin	2017-11-2	49,693	71	45
SAMN26027622	SRR18111937	Wisconsin	2016-10-5	50,908	71	55
SAMN26027623	SRR18111936	Wisconsin	2017-10-4	47,985	59	37
SAMN26027624	SRR18111935	Wisconsin	2016-9-21	36,116	59	49
SAMN26027625	SRR18111934	Wisconsin	2017-9-26	41,013	80	59

### Data availability.

Demultiplexed FASTQ files can be found in the NCBI Sequence Read Archive (SRA) under BioProject accession number PRJNA809416. Annotated files, additional analyses, and code are available at GitHub (https://github.com/NewtonLabUWM/Full16S_sewageDatabase).
